# Evaluation of a telethermographic system for temperature screening at a large tertiary-care referral hospital during the coronavirus disease 2019 (COVID-19) pandemic

**DOI:** 10.1017/ice.2020.1254

**Published:** 2020-10-12

**Authors:** Katherine C. Leach, Misti G. Ellsworth, Luis Z. Ostrosky, Cynthia S. Bell, Kathy Masters, John Calhoun, Lance Ferguson, Susie Distefano, Michael L. Chang

**Affiliations:** 1 Department of Pediatrics, UTHealth McGovern Medical School, Houston, Texas; 2 Department of Internal Medicine, UTHealth McGovern Medical School, Houston, Texas; 3 Memorial Hermann Hospital, Texas Medical Center, Houston, Texas

Since the outset of the coronavirus disease 2019 (COVID-19) pandemic, the United States Centers for Disease Control and Prevention has recommended screening and triage for signs and symptoms of infection for everyone entering a healthcare facility.^1^ In compliance, everyone entered our hospital (a tertiary-care referral center in a large metropolitan area) in several single-file lines, and underwent individual symptom screening and temperature check by temporal artery thermometer that required skin contact and cleaning of the probe cone between uses. Despite optimizations, temperature screening resulted in long lines during employee shift changes, which compromised social distancing and exposed screeners to hundreds of individuals in close proximity. During this period, the US Food and Drug Administration issued guidance for initial temperature assessment during a triage process using telethermographic systems (thermal cameras) able to determine surface skin temperature from a distance without skin contact.^2^

We sought to determine the feasibility of replacing temporal artery thermometers with a telethermographic system and the impact of such a system on our screening process.

## Methods

Temperatures were measured with TAT-2000 and TAT-5000 TemporalScanner thermometers (Exergen Corp, Watertown, MA) and the Athena Elevated Temperature Detection System (Athena Security, Austin, TX). Exergen reports their instruments to be accurate within 0.2°C and 0.1°C, respectively.^3,4^ The Athena telethermographic system uses artificial intelligence to detect human faces by measuring the temperature of multiple points on the face relative to a blackbody temperature reference source.^5^ According to Athena Security, the system is accurate within 0.3°C.^5^ Systems were purchased from Athena Security.

Accepting manufacturer specifications, detecting 0.2°C difference between devices (assuming standard deviation of ±0.3°C) required 26 measurements from each device. One subject was measured 104 times with 4 different TAT-2000s (26 measurements per device) and 104 times with 4 different TAT-5000s (26 measurements per device) by a single operator, and 13 times with the Athena system at a single location within 90 minutes to minimize subject and environmental temperature variation. We repeated measurements with the same subject and thermometer operator at a second location with 3 additional TAT-5000s, 1 TAT-5000 used previously (104 measurements, 26 per device) and a second thermal camera (13 measurements). We simulated fever using air-activated hand warmers (HotHands, Kobayashi Americas, Dalton, GA) held to the forehead. Descriptive statistical analyses were performed with Stata version 15 SE software (StataCorp, College Station, TX). Summaries were reported as means with 95% confidence intervals and differences were tested by 1-way ANOVA. A 2-sided *P* < .05 was considered statistically significant.

## Results

### Temperature measurement

During the first session, the TAT-2000s measured higher temperatures [mean, 98.3°F (95% CI, 98.2–98.3) or 36.8°C (95% CI, 36.8–36.8)] than the TAT-5000s [mean, 97.8 °F (95% CI, 97.8–97.9) or 36.6°C (95% CI, 36.5–36.6)] or the Athena system [mean, 97.9°F (95% CI, 97.8–98.0) or 36.6°C (95% CI, 36.5–36.7)] (*P* < .05). There was no significant difference between the TAT-5000s and the Athena system [mean difference, −0.07°F (95% CI, −0.23 to 0.09) or −0.04°C (95% CI, −0.13 to 0.05)], but the TAT-2000s measured temperatures significantly higher than the Athena [mean difference, 0.40°F (95% CI, 0.24–0.56) or 0.22°C (95% CI, 0.13–0.31)]. During the second testing session, the TAT-5000s measured 0.34°F (95% CI, 0.20–0.48) or 0.19°C (95% CI, 0.11–0.26) [mean, 98.1°F (95% CI, 98.1–98.2) or 36.7°C (95% CI, 36.7–36.8)] higher than the Athena system [mean, 97.8°F (95% CI, 97.7–97.9) or 36.6°C (95% CI, 36.5–36.6)] (*P* < .05).

### Fever detection by the Athena system

**Fig. 1.**
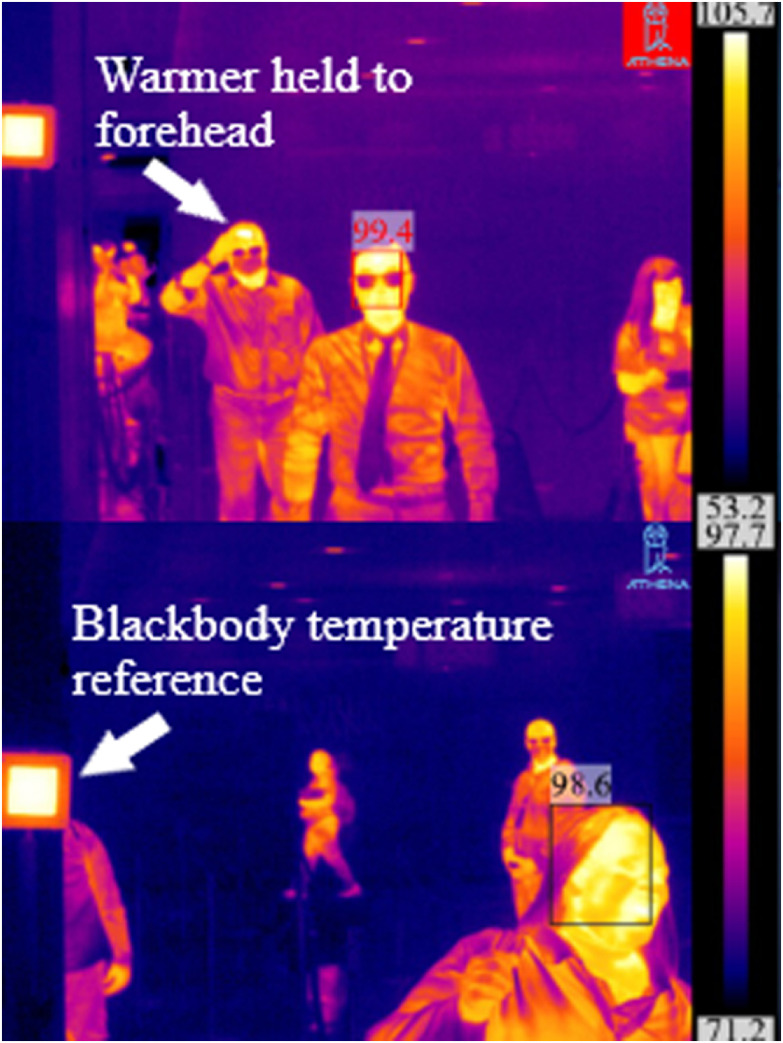
(Top) Initial test run for fever detection by telethermographic system. Individuals passed camera in single-file line at rate of 1 individual per second ~6 feet (~2 m) apart with warmers held to forehead and then removed. In total, 13 tests were then conducted as described. Maximum temperature detected in frame of 105.7°F (40.94°C) identified as a warmer. (Bottom) Facial detection, normal temperature, and location of black body temperature reference within the camera frame.

HotHands warmers reached up to 46.1°C (115°F) 15–30 minutes after activation and were held at the forehead. A “symptomatic” individual in single-file line 6 feet (2 m) between “normal” individuals passing the camera at a rate of 1 individual per second, was detected in 8 of 8 attempts. Additionally, when the forehead was warmed and the warmer then removed, the Athena system was able to detect temperatures of >99°F or 37.2°C (Fig. 1) in 5 of 5 attempts.

### Screening time

Screeners using TAT-5000s took a median of 16.5 seconds from the start of taking the temperature through cleaning the device until the thermometer was ready again. The Athena system has no effective delay from person to person passing in a single-file line.

## Discussion

The COVID-19 pandemic has led to the implementation of temperature screening in a wide variety of facilities. Although temperature screening has been used in public settings during previous infectious diseases outbreaks,^6–8^ the usefulness of temperature screening to detect potential infections has been questioned.^6,7^ However, temperature screening may discourage symptomatic individuals from entering public places and may increase comfort for healthy people.^8^

Our study using noninvasive devices was not designed to test the accuracy of devices, though temporal scanners are widely considered reliable enough for professional use.^9,10^ In our use, temperatures measured by telethermographic systems were similar to those obtained by temporal scanners, suggesting similar performance.

Cost is the biggest barrier to implementation for telethermographic systems. For our investment recovery analysis, we considered turnaround time difference between temporal scanners and a thermal camera for each screened individual at a high-entry location with large groups arriving in a short period, desired throughput rate of 1 person per second, maintaining 6-feet (2-m) social distancing and single-file lines for individual symptom screening. We estimated needing 6 temporal scanner operators for every 1 thermal camera operator. With our organization’s direct labor rates and overhead costs, investment recovery was estimated to occur in months, leading to adoption of 4 telethermographic systems at our 2 highest-entry locations. We reduced screening staff from 24 to 4 individuals, and there are now no waiting lines at these locations.

In conclusion, our experience demonstrates that a telethermographic system improves screening throughput and reports temperatures similar to those recorded by temporal scanners, with acceptable investment recovery time.
